# Effects of endurance training on metabolic enzyme activity and transporter protein levels in the skeletal muscles of orchiectomized mice

**DOI:** 10.1186/s12576-022-00839-z

**Published:** 2022-06-29

**Authors:** Kenya Takahashi, Yu Kitaoka, Hideo Hatta

**Affiliations:** 1 Department of Sports Sciences, Graduate School of Arts and Sciences, The University of Tokyo, 3-8-1, Komaba, Meguro-ku, Tokyo, 153-8902 Japan; 2 Department of Human Sciences, Kanagawa University, 3-27-1, Rokkakubashi, Kanagawa-ku, Yokohama, Kanagawa 221-8686 Japan

**Keywords:** Orchiectomy, Exercise, Enzyme, Transporter, Skeletal muscle

## Abstract

This study investigated whether endurance training attenuates orchiectomy (ORX)-induced metabolic alterations. At 7 days of recovery after sham operation or ORX surgery, the mice were randomized to remain sedentary or undergo 5 weeks of treadmill running training (15–20 m/min, 60 min, 5 days/week). ORX decreased glycogen concentration in the gastrocnemius muscle, enhanced phosphofructokinase activity in the plantaris muscle, and decreased lactate dehydrogenase activity in the plantaris and soleus muscles. Mitochondrial enzyme activities and protein content in the plantaris and soleus muscles were also decreased after ORX, but preserved, in part, by endurance training. In the treadmill running test (15 m/min, 60 min) after 4 weeks of training, orchiectomized sedentary mice showed impaired exercise performance, which was restored by endurance training. Thus, endurance training could be a potential therapeutic strategy to prevent the hypoandrogenism-induced decline in muscle mitochondrial content and physical performance.

## Background

Androgens are male sex hormones that are secreted predominantly from the testes. Androgen deficiency or insufficiency occurs under several conditions, such as aging and disease [1], leading to negative health consequences [2]. Circulating testosterone levels are positively correlated with insulin sensitivity, maximal oxygen uptake, and mitochondrial gene expression [3], suggesting that bioavailable androgen levels are closely associated with metabolic homeostasis, whole-body metabolism, and physical performance.

Although androgen restoration is a treatment option for men with hypogonadism, several adverse effects have been reported. For example, a study conducted in old frail men with a high prevalence of cardiovascular diseases was halted prematurely due to high rates of cardiac, respiratory, and dermatologic events in the treatment group [4]. Therefore, an alternative approach to hypoandrogenism is warranted. Exercise training prevents several metabolic disorders, by potentially improving the metabolic capacity of skeletal muscles [5, 6]. However, it remains unclear whether endurance training is a viable strategy to alleviate androgen deficiency-induced metabolic impairments, and whether loss of androgens impedes skeletal muscle adaptation to endurance training.

In this study, we examined the effects of endurance training on glycolytic and oxidative enzyme activities in the skeletal muscles of mice that underwent orchiectomy (ORX) surgery, which is a prevailing model of androgen deficiency. Since substrate metabolism is regulated by transport activity at the plasma membrane, we also determined the protein levels of key metabolite transporters. Given that androgen sensitivity is likely to differ depending on the muscle phenotype [7–9], we analyzed the plantaris (glycolytic phenotype) and soleus (oxidative phenotype) muscles. Moreover, we performed respiratory gas analysis during exercise to evaluate whole-body metabolism. Given that androgen level is reported to associate with metabolic function [3], we hypothesized that enzyme activity and transport protein levels are declined by ORX, but restored by endurance training.

## Methods

### Animals

All experiments were approved by the Animal Experimental Committee of The University of Tokyo (No. 2021-1). Ten-week-old male Institute of Cancer Research (ICR) mice bred in the animal care facility at The University of Tokyo were used in this study. The animals were housed individually on a 12:12 h light/dark cycle (dark: 7:00 to 19:00) in an air-conditioned room (23 °C). All mice had ad libitum access to standard chow diet (Oriental Yeast, Tokyo, Japan) and water during the experimental period.

### Experimental design

**Fig. 1:**
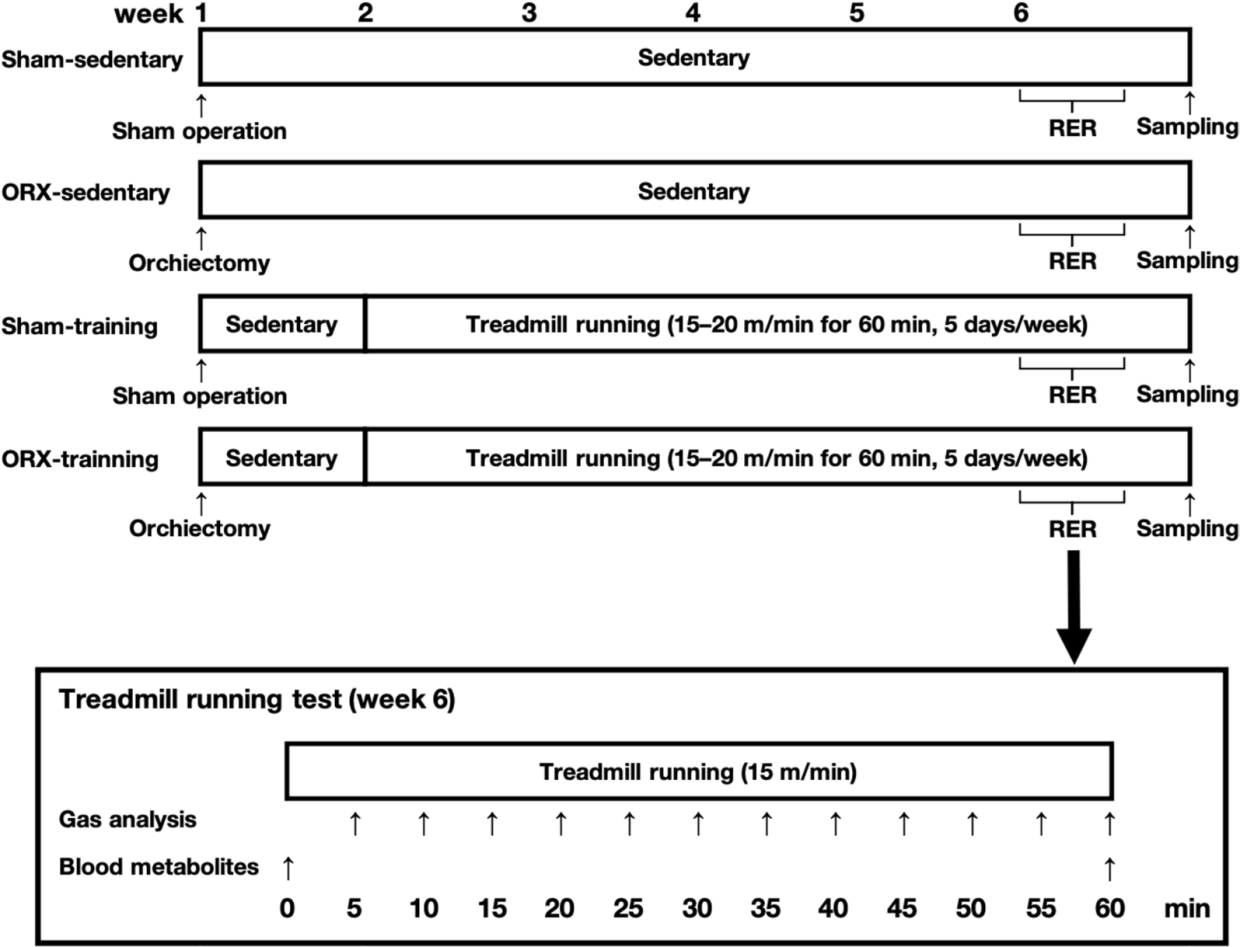
Schematic overview of the experiment

Figure 1 shows the schematic of the experiment. Before the experiment, all animals were familiarized with running on a treadmill (MK-680; Muromachi Kikai Co., Inc., Tokyo, Japan) at a speed of 20 m/min for 5 min for 3 days. At 10 weeks of age, the animals underwent a sham operation or ORX surgery, as described below. Following a 7-day post-surgery recovery, animals were subdivided into sedentary and training groups as follows: sham-sedentary group (*n* = 9), ORX-sedentary group (*n* = 10), sham-training group (*n* = 8), and ORX-training group (*n* = 11). Animals in the training groups performed 60 min of treadmill running 5 days a week for 5 weeks. According to a previous study, the critical speed, where the greatest metabolic rate that results in wholly oxidative energy provision is represented [10], for ICR mice is 24.1 ± 4.6 m/min [11]. In our previous study, we used treadmill running at 20 m/min for 60 min as endurance training for ICR mice [12]. In our preliminary study, however, we observed that animals were unable to complete 60 min of exercise at 20 m/min, while they were able to complete it at 15 m/min after a 7-day post-surgery. Therefore, the running speed for the initial five training sessions was set at 15 m/min, followed by 20 m/min for subsequent training sessions. The treadmill running test was performed 4–7 days before tissue sampling to avoid exercise effects on the sedentary groups, as described below. Twenty-four hours after the last training session, the animals were anesthetized using isoflurane and euthanized by removing blood from the inferior vena cava. The gastrocnemius, plantaris, and soleus muscles were collected, rapidly frozen in liquid nitrogen, and stored at − 80 °C until analysis.

### ORX surgery

The animals were anesthetized via an intraperitoneal injection of a cocktail (5 µL/g body weight) of medetomidine hydrochloride (0.3 µg/g body weight), midazolam (4.0 µg/g body weight), and butorphanol (5.0 µg/g body weight). Small incisions (~ 0.5 cm) were made on both sides of the scrotum. The bilateral testes were removed from the incisions and excised, leaving the epididymal fat pad and seminal vesicles in place. An identical procedure was performed in the sham-operated groups, except that the testes were left intact. The incisions were closed using a surgical needle and a 3–0 absorbable suture. After all surgical procedures were completed within 10 min, atipamezole hydrochloride (3.0 µg/g body weight, 5 µL/g body weight) was intraperitoneally administered to negate anesthesia.

### Treadmill running test

The animals performed treadmill running at a speed of 15 m/min for 60 min in an airtight metabolic chamber equipped with a treadmill (MK-680AT/02M; Muromachi Kikai). This treadmill running speed is considered lower than the critical speed for typical ICR mice [11]. O_2_ consumption (VO_2_) and CO_2_ production (VCO_2_) were measured every 5 min using a metabolism-measuring system (MK-5000RQ; Muromachi Kikai) with an airflow rate of 1.5 L/min. The respiratory exchange ratio (RER) was calculated as VCO_2_/VO_2_. The measurement was terminated when the animals reached exhaustion before 60 min of exercise. Exhaustion was defined as the inability of the animals to maintain the running speed despite contacting the electrical grid for more than 5 consecutive seconds. Before and after exercise, the tail vein blood glucose and lactate levels were measured using GLUCOCARD Plus Care (Arkray, Kyoto, Japan) and Lactate Pro 2 (Arkray), respectively.

### Muscle glycogen

The glycogen content in the gastrocnemius muscle was measured as previously described [13]. Briefly, the whole gastrocnemius muscle was heated at 100 °C in 30% (w/v) KOH solution saturated with Na_2_SO_4_ until completely dissolved. Glycogen in the solution was precipitated on ice for 30 min after the addition of 99% (v/v) ethanol. The solution was then centrifuged at 10,000×*g* for 10 min at 4 °C. After the supernatant was discarded, the glycogen precipitate was dissolved in 1 N HCl and heated at 100 °C for 2 h to hydrolyze glycogen to glucose. After neutralization with 1 N NaOH, glucose concentration was determined using a glucose CII kit (Fujifilm Wako, Osaka, Japan).

### Muscle triglyceride (TG)

Whole gastrocnemius muscle was homogenized using a µT-01 bead crusher (TAITEC, Saitama, Japan) in a buffer containing 5% (v/v) NP-40 substitute (145-09701; Fujifilm Wako). The homogenate was subjected to two cycles of heating (100 °C for 5 min) and cooling (room temperature) to solubilize the lipids. After centrifugation at 10,000×*g* for 2 min, the TG content in the supernatant was determined using a LabAssay Triglyceride kit (Fujifilm Wako).

### Determination of enzyme activity

Whole soleus and plantaris muscles were homogenized in 100 times (vol/wt) of phosphate buffer (100 mM, pH 7.6) using a μT-01 bead crusher (TAITEC). The homogenates were freeze-thawed twice using liquid nitrogen to disrupt the plasma and mitochondrial membranes. After centrifugation at 1000×*g* for 10 min at 4 °C, the supernatant was recovered for the enzyme assay.

#### Hexokinase (HK) assay

Maximal HK activity was measured as previously described, with slight modifications [14]. The aliquots were mixed with the reaction mixture (50 mM triethanolamine, 5 mM EDTA, 10 mM MgCl_2_, 0.35 mM NADH, 2.8 mM ATP, 2.8 mM glucose, and 2.5 U glucose-6-phosphatase, pH 7.6) in a 96-well microplate (195-96F; Watson Bio Lab, Tokyo, Japan). The changes in absorbance at 340 nm were determined using a microplate spectrophotometer (Epoch Microplate Spectrophotometer, BioTek Instruments, Inc.).

#### Phosphofructokinase (PFK) assay

Maximal PFK activity was measured as previously described, with slight modifications [14]. The aliquots were mixed with the reaction mixture (50 mM triethanolamine, 5 mM EDTA, 10 mM MgCl_2_, 0.3 mM NADH, 2.8 mM ATP, 2.8 mM F-6-P, 2.5 U GPDH-TPI, and 1.0 U aldolase, pH 7.6) in a 96-well microplate. The changes in absorbance at 340 nm were determined.

#### Lactate dehydrogenase (LDH, pyruvate-to-lactate) assay

The activity of LDH, which involved the conversion of pyruvate to lactate, was measured as previously described, with slight modifications [15]. The aliquots were mixed with the reaction mixture (50 mM imidazole, 5 mM DTT, 150 μM NADH, 4.0 mM pyruvate, pH 7.4) in a 96-well microplate. The changes in absorbance at 340 nm were determined.

#### Citrate synthase (CS) assay

Maximal CS activity was measured as previously described, with slight modifications [16]. The aliquots were mixed with the reaction mixture (100 mM Tris, 100 μM DTNB, 300 μM acetyl-CoA, and 50 μM oxaloacetate, pH 8.3) in a 96-well microplate. The changes in absorbance at 412 nm/min were determined.

#### Cytochrome *c* oxidase (COX) assay

Maximal COX activity was measured as previously described, with slight modifications [17]. The aliquots were mixed with the reaction mixture (10 mM phosphate and 50 μM cytochrome *c* reduced with sodium hydrosulfite, pH 7.0) in a 96-well microplate. The changes in absorbance at 550 nm/min were determined.

#### β-Hydroxyacyl-CoA dehydrogenase (β-HAD) assay

The maximal β-HAD activity was measured as previously described, with slight modifications [18]. The aliquots were mixed with the reaction mixture (1 M Tris, 5 mM EDTA, 450 µM NADH, and 100 µM acetoacetyl-CoA, pH 7.0) in a 96-well microplate. The changes in absorbance at 340 nm/min were determined.

#### Total carnitine palmitoyltransferase (CPT) assay

The maximal activity of CPT (CPT-I and CPT-II) was measured as described previously, with slight modifications [19]. The aliquots were mixed with the reaction mixture (60 mM Tris, 1.5 mM EDTA, 0.25 mM DTNB, 1.67 mM l-carnitine, and 0.025 mM palmitoyl-CoA, pH 8.0) in a 96-well microplate. The changes in absorbance at 412 nm/min were determined.

### Western blotting

Whole soleus and plantaris muscles were homogenized 20 times (vol/wt) in ice-cold radioimmunoprecipitation assay buffer (25 mM Tris-HCl, pH 7.6, 150 mM NaCl, and 1% NP-40) supplemented with a protease inhibitor cocktail (cOmplete Mini, ETDA-free; Roche Applied Science, Indianapolis, IN, USA) using a μT-01 bead crusher (TAITEC). The homogenates were rotated on ice for 60 min and centrifuged at 1500×*g* at 4 °C for 20 min. The total protein content of the samples was determined using a BCA protein assay kit (TaKaRa BIO Inc., Shiga, Japan). Equal amounts of proteins were loaded onto sodium dodecyl sulfate–polyacrylamide gels and separated via electrophoresis. Proteins were transferred onto polyvinylidene difluoride membranes and western blotting was performed using the standard procedure, as previously described [20]. The primary and secondary antibodies used in this study are mentioned below. Blots were scanned and quantified using ChemiDoc XRS (Bio-Rad Laboratories, Hercules, CA, USA) and Quantity One (version 4.5.2; Bio-Rad). Ponceau staining was used to verify the consistent loading.

### Primary and secondary antibodies

Commercially available primary antibodies were used to detect hypoxia-inducible factor 1-α (HIF-1α; #20960-1-AP; Proteintech Japan, Tokyo, Japan), peroxisome proliferator-activated receptor γ coactivator 1-α (PGC-1α; #516557; Merck Millipore), mitochondrial electron transport proteins (NADH:ubiquinone oxidoreductase subunit B8 [NDUFB8], succinate dehydrogenase complex iron sulfur subunit B [SDHB], ubiquinol–cytochrome *c* reductase core protein 2 [UQCRC2], mitochondrially encoded cytochrome c oxidase 1 [MTCO1], and ATP synthase F1 subunit alpha [ATP5F1A/ATP5A]; #ab110413; Abcam, Cambridge, UK), cytochrome *c* oxidase subunit 4I1 (COX4I1/COXIV; #ab14744; Abcam), glucose transporter 1 (GLUT1; #sc-377228; Santa Cruz Biotechnology, Santa Cruz, CA, USA), glucose transporter 4 (GLUT4; #07-1404; Merck Millipore, Tokyo, Japan), and fatty acid translocase/cluster of differentiation 36 (FAT/CD36; #18836-1-AP; Proteintech Japan). Antibodies against monocarboxylate transporter (MCT)-1 and MCT-4 were raised in rabbits against the C-terminal region of the respective MCT (Qiagen, Tokyo, Japan), and have been used in our previous studies [13, 21–23]. Rabbit anti-goat IgG (H&L) (#A102PT; American Qualex, San Clemente, CA, USA) and mouse anti-goat IgG (H&L) (#A106PU; American Qualex) were used as secondary antibodies.

### Statistical analysis

All data are presented as the mean ± standard error of the mean. Two-way analysis of variance (ANOVA) was applied to determine the interaction and main effects of training and ORX on mice. When an interaction was significant, a comparison was made using the Tukey–Kramer multiple comparison test to identify differences among the groups. The running proportion curves in the treadmill running test were compared using the log-rank (Mantel–Cox) test. For the time course changes in RER, two-way ANOVA (time × group) followed by the Tukey–Kramer multiple comparison test was performed. All statistical analyses were performed using the GraphPad Prism software (Ver. 9.0, Macintosh; GraphPad Software, La Jolla, CA). Statistical significance was defined as *p* < 0.05. All results within the range of 0.05 ≤ *p* ≤ 0.1 were shown as tendencies.

## Results

### Body and tissue weights, and food intake

**Fig. 2:**
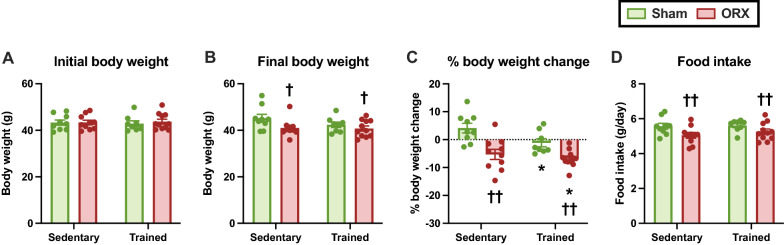
Body weight and food intake. Initial (**A**) and final (**B**) body weights. Body weight change (**C**) and food intake (**D**) during the experimental period. Data are expressed as the mean ± standard error of the mean (SEM) (*n* = 8–11). Two-way analysis of variance (ANOVA) was performed to determine the interactions and main effects of training and ORX. *p < 0.05: main effect of training. ^††^*p* < 0.01, ^†^*p* < 0.05: main effect of ORX

**Fig. 3:**
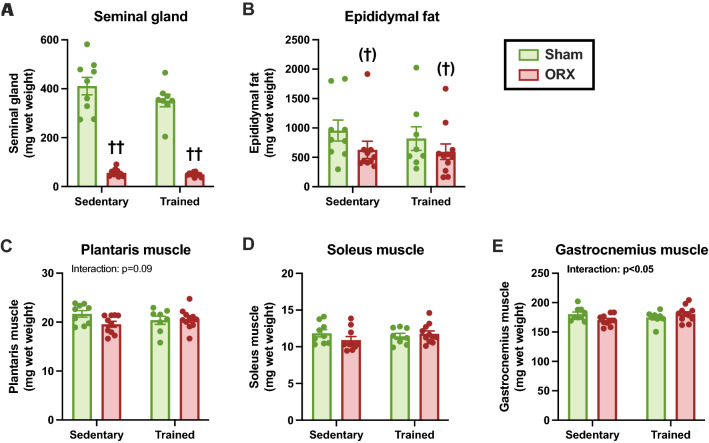
Tissue weights after the experimental period. **A** Seminal vesicle and **B** epididymal fat weights. Plantaris (**C**), soleus (**D**), and gastrocnemius (**E**) muscle weights. Data are expressed as the mean ± SEM (*n* = 8–11). Two-way ANOVA was performed to determine the interactions and main effects of training and ORX. ^††^*p* < 0.01, ^(†)^*p* ≤ 0.10: main effect of ORX

There were no differences in the initial body weights among the groups (Fig. 2A); however, ORX resulted in the lower final body weight (*p* < 0.05, Fig. 2B). We also found that ORX (*p* < 0.05) and training (*p* < 0.01) reduced the body weight during the experimental period (Fig. 2C). ORX decreased food intake during the experimental period (*p* < 0.01, Fig. 2D), suggesting that the lower final body weight was, in part, due to a decline in food consumption. ORX dramatically decreased seminal vesicle weight (*p* < 0.01, Fig. 3A), suggesting the successful removal of testes and a reduction in bioavailable testosterone after ORX. There was a trend for lower epididymal fat weight after ORX administration (*p* = 0.10, Fig. 3B). The plantaris, soleus, and gastrocnemius muscle weights did not differ significantly among the groups (Fig. 3C–E). These observations suggest that the decline in body weight after ORX is likely to result from a reduction in body fat mass caused by decreased food intake. Moreover, ORX-induced atrophy of androgen-sensitive tissues may partially account for body weight loss, as evidenced by the significant decrease in the seminal vesicle weight.

### Treadmill running test

**Fig. 4:**
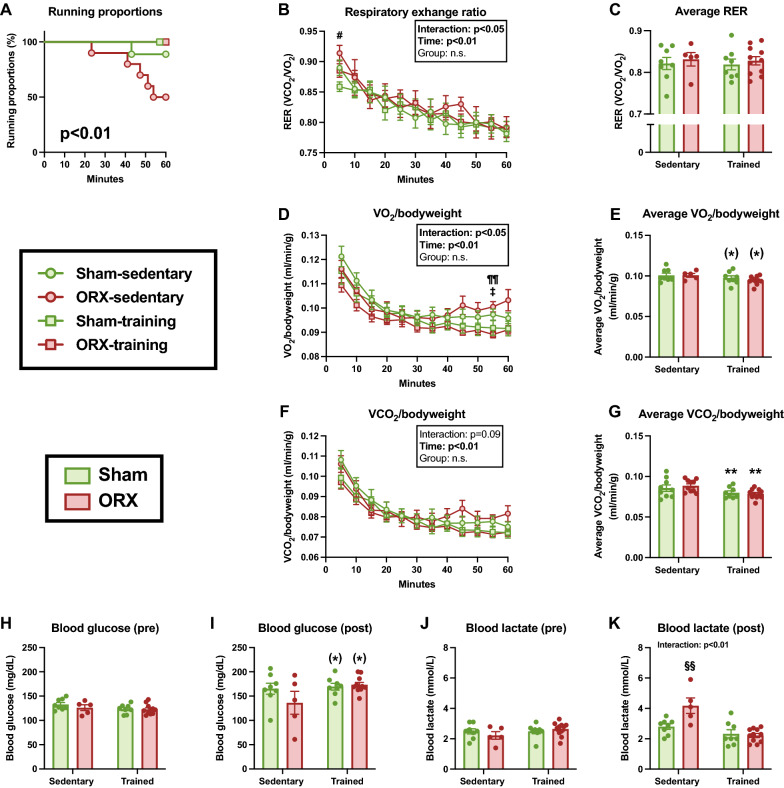
Respiratory exchange ratio and blood metabolite levels in the treadmill running test. Running proportions (**A**), time-course changes in the respiratory exchange ratio (RER) (**B**), and average RER (**C**) during 60 min of treadmill running. Time-course changes in O_2_ consumption (VO_2_)/bodyweight (**D**), and average VO_2_/bodyweight (**E**) during 60 min of treadmill running. Average RER (**C**) during 60 min of treadmill running. Time-course changes in CO_2_ production (VCO_2_)/bodyweight (**F**), and average VCO_2_/bodyweight (**G**) during 60 min of treadmill running. Blood glucose concentrations before (**H**) and after (**I**) exercise. Blood lactate concentrations before (**J**) and after (**K**) exercise. Data are expressed as the mean ± SEM (*n* = 5–11). The running proportion curves in the treadmill running test were compared using the log-rank (Mantel–Cox) test. For the time course changes in RER, two-way ANOVA (time × group) followed by the Tukey–Kramer multiple comparison test was performed. To determine the interactions and main effects of training and ORX, two-way ANOVA followed by the Tukey–Kramer multiple comparison test was performed. ^#^*p* < 0.05: ORX-sedentary group vs. sham-training group. ^‡^*p* < 0.05: sham-sedentary group vs. ORX-training group. ^¶¶^*p* < 0.01: ORX-sedentary group vs. ORX-training group. **p < 0.01, ^(^*^)^*p* ≤ 0.10: effect of training. ^§§^*p* < 0.01: significantly different from other groups

To elucidate the impact of ORX on whole-body metabolism during exercise, we performed gas analysis during treadmill running 5 weeks after surgery. During this test, one mouse in the sham-sedentary group and five mice in the ORX-sedentary group reached exhaustion before 60 min, generating significant differences in the running proportion curves (*p* < 0.01, Fig. 4A). As they could not complete 60 min of exercise, we excluded their respiratory and blood metabolite data from the statistical analysis. Although the RER at 5 min of treadmill running was significantly higher in the ORX-sedentary group than in the sham-training group (*p* < 0.05, Fig. 4B), no significant differences were observed in the average RER (Fig. 4C). VO_2_/bodyweight at 55 min was significantly higher in the ORX-sedentary group than in the sham-training (*p* < 0.05) and ORX-training groups (*p* < 0.01, Fig. 4D). In the time course changes in VCO_2_/bodyweight, we observed a main effect of time (*p* < 0.01), but not a main effect of ORX or significant interaction (*p* = 0.09; Fig. 4F). Endurance training tended to decrease average VO_2_/bodyweight (*p* = 0.05, Fig. 4E), and significantly decreased VCO_2_/bodyweight (*p* < 0.01, Fig. 4G). The blood lactate and glucose levels before exercise were not significantly different (Fig. 4H, J). There was no significant difference in the post-exercise glucose concentration (Fig. 4I). Blood lactate levels after 60 min of exercise were significantly higher in the ORX-sedentary group than in the other groups (*p* < 0.01; Fig. 4K). These observations suggest that ORX impairs the endurance exercise performance. However, this impairment is likely to be restored by endurance training.

### Glycogen and TG levels

**Fig. 5:**
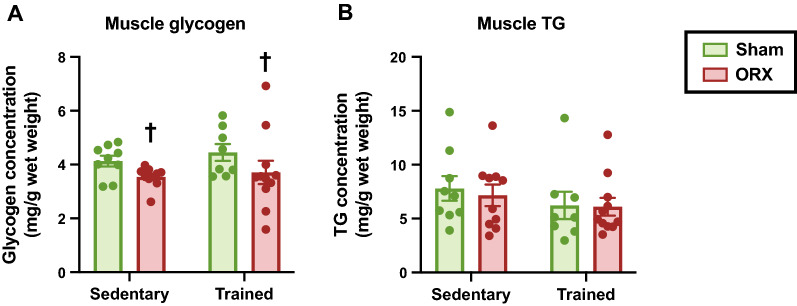
Muscle glycogen and triglyceride (TG) concentrations. Glycogen (**A**) and TG (**B**) concentrations in the gastrocnemius muscle at rest. Data are expressed as the mean ± SEM (*n* = 8–11). Two-way ANOVA was performed to determine the interactions and main effects of training and ORX. ^†^*p* < 0.05: main effect of ORX

Given the impaired exercise performance of ORX-sedentary animals, we evaluated the basal level of muscle glycogen, which is a determinant factor of prolonged exercise performance [24]. We found that ORX reduced glycogen concentration in the gastrocnemius muscle at rest (*p* < 0.05, Fig. 5A), suggesting that a lower abundance of muscle glycogen was partially responsible for the compromised performance. We also determined the level of TG, which is another energy deposit stored in the skeletal muscle. No significant effect was detected on the TG concentration in the gastrocnemius muscle (Fig. 5B).

### Glycolytic enzyme activity

**Fig. 6:**
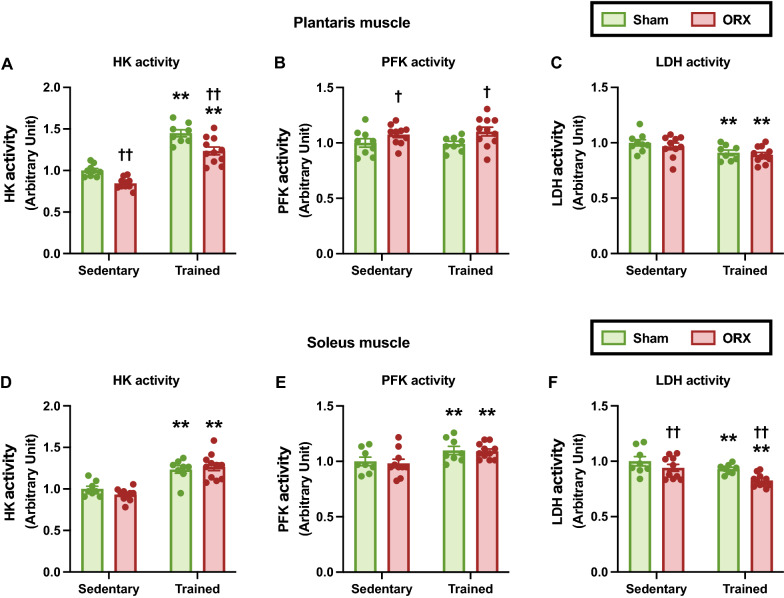
Glycolytic enzyme activities. Hexokinase (HK) (**A**), phosphofructokinase (PFK) (**B**), and lactate dehydrogenase (LDH) (**C**) activities in the plantaris muscle. HK (**D**), PFK (**E**), and LDH (**F**) activities in the soleus muscle. Data are expressed as the mean ± SEM (*n* = 8–11). Two-way ANOVA was performed to determine the interactions and main effects of training and ORX. **p < 0.01: main effect of training. ^††^*p* < 0.01, ^†^*p* < 0.05: main effect of ORX

To clarify the metabolic characteristics of skeletal muscles, we first evaluated the glycolytic enzyme activity. Endurance training significantly enhanced HK activity in the plantaris (*p* < 0.01, Fig. 6A) and soleus (*p* < 0.01, Fig. 6D) muscles and PFK activity in the soleus muscle (*p* < 0.01, Fig. 6E). In addition, endurance training decreased LDH activity in the plantaris (*p* < 0.01, Fig. 6C) and soleus (*p* < 0.01, Fig. 6F) muscles. ORX increased PFK activity (*p* < 0.05, Fig. 6B) and decreased HK activity (*p *< 0.01, Fig. 6A) in the plantaris muscle and LDH activity in the soleus muscle (*p* < 0.01, Fig. 6F). These observations suggest that ORX can change the glycolytic capacity of skeletal muscles.

### Transcription factors involved in glycolytic metabolism

**Fig. 7:**
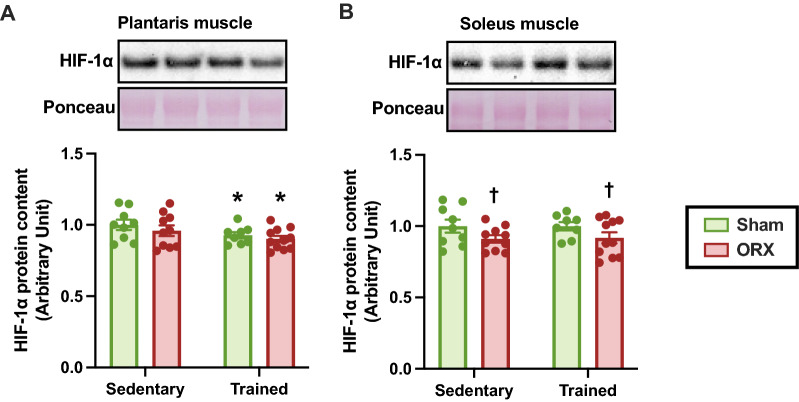
Hypoxia-inducible factor 1-α (HIF-1α) protein levels. HIF-1α protein levels in the plantaris (**A**) and soleus (**B**) muscles. Data are expressed as the mean ± SEM (*n* = 8–11). Two-way ANOVA was performed to determine the interactions and main effects of training and ORX. *p < 0.05: main effect of training. ^†^*p* < 0.05: main effect of ORX

To explore the potential mechanisms by which glycolytic enzyme activity is altered, we determined the protein levels of HIF-1α, a transcription factor that regulates glycolytic metabolism. Endurance training significantly reduced HIF-1α protein levels in the plantaris muscle (*p* < 0.05, Fig. 7A). In soleus muscle, HIF-1α protein content was decreased by ORX (*p* < 0.05, Fig. 7B). These observations suggest that HIF-1α protein levels do not always correlate with changes in glycolytic enzyme activity.

### Mitochondrial enzyme activity

**Fig. 8:**
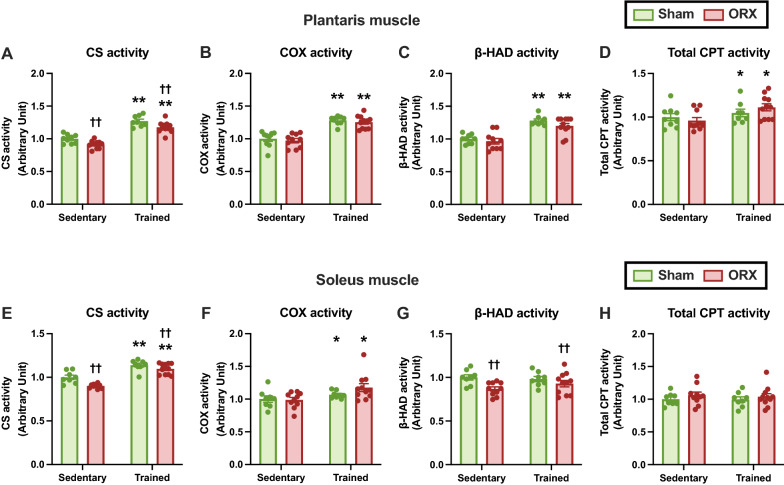
Mitochondrial enzyme activities. Citrate synthase (CS) (**A**), cytochrome *c* oxidase (COX) (**B**), β-hydroxyacyl-CoA dehydrogenase (β-HAD) (**C**), and total carnitine palmitoyltransferase (CPT) (**D**) activities in the plantaris muscle. CS (**E**), COX (**F**), β-HAD (**G**), and total CPT (**H**) activities in the soleus muscle. Data are expressed as the mean ± SEM (*n* = 8–11). Two-way ANOVA was performed to determine the interactions and main effects of training and ORX. **p < 0.01, *p < 0.05: main effect of training. ^††^*p* < 0.01: main effect of ORX

Next, we assessed the enzyme activities of the mitochondria, which are the key components for energy production. Endurance training enhanced CS and COX activity in the plantaris (*p* < 0.01, Fig. 8A, B) and soleus (*p* < 0.01, Fig. 8E; *p* < 0.05, Fig. 8F) muscles, as well as β-HAD (*p* < 0.01, Fig. 8C) and total CPT activity (*p* < 0.05, Fig. 8D) in the plantaris muscle. ORX significantly reduced CS activity in the plantaris (*p* < 0.01, Fig. 8A) and soleus (*p* < 0.01, Fig. 8E) muscles and β-HAD activity in the soleus muscle (*p* < 0.01, Fig. 8G). There was no significant difference in the total CPT activity of the soleus muscle (Fig. 8H). Thus, the mitochondrial enzyme activity is likely to decline after ORX.

### Mitochondria-associated proteins

**Fig. 9:**
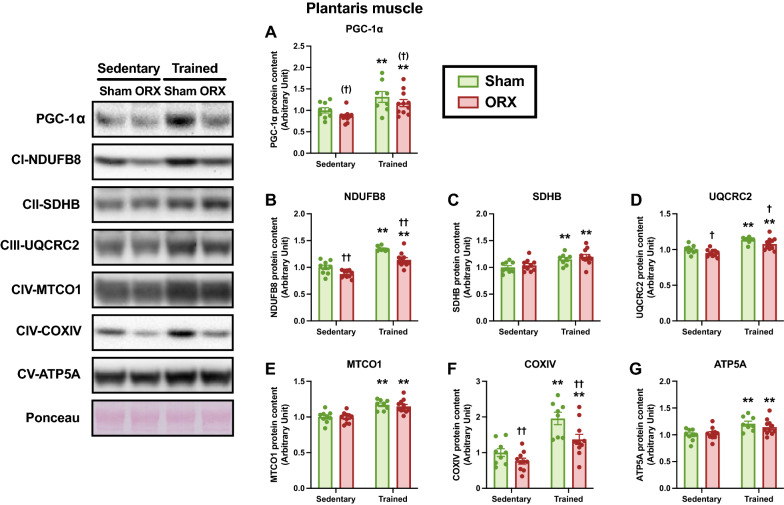
Mitochondria-associated protein levels in the plantaris muscle. Protein levels of peroxisome proliferator-activated receptor γ coactivator 1-α (PGC-1α) (**A**), NADH:ubiquinone oxidoreductase subunit B8 (NDUFB8) (**B**), succinate dehydrogenase complex iron sulfur subunit B (SDHB) (**C**), ubiquinol–cytochrome *c* reductase core protein 2 (UQCRC2) (**D**), mitochondrially encoded cytochrome c oxidase 1 (MTCO1) (**E**), cytochrome *c* oxidase subunit 4I1 (COX4I1/COXIV) (**F**), and ATP synthase F1 subunit alpha (ATP5F1A/ATP5A) (**G**) in the plantaris muscle. Data are expressed as the mean ± SEM (*n* = 8–11). Two-way ANOVA was performed to determine the interactions and main effects of training and ORX. **p < 0.01, *p < 0.05: main effect of training. ^††^*p* < 0.01, ^(†)^*p* ≤ 0.10: main effect of ORX

**Fig. 10:**
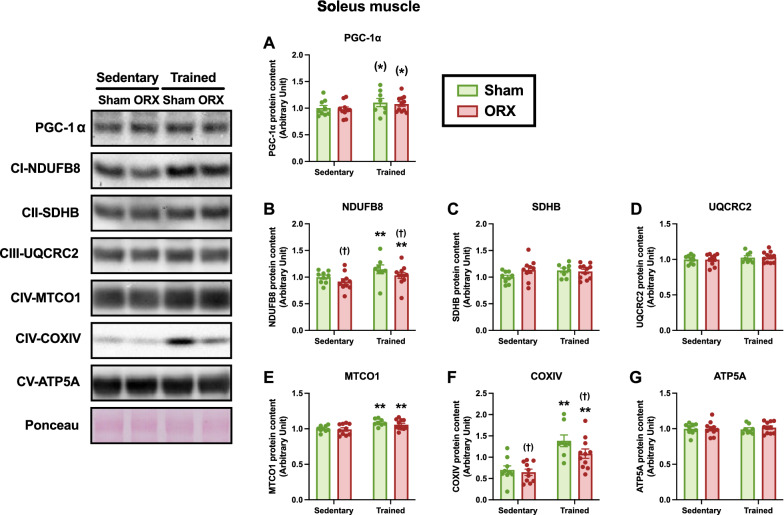
Mitochondria-associated protein levels in the soleus muscle. Protein levels of PGC-1α (**A**), NDUFB8 (**B**), SDHB (**C**), UQCRC2 (**D**), MTCO1 (**E**), COXIV (**F**), and ATP5A (**G**) in the soleus muscle. Data are expressed as the mean ± SEM (*n* = 8–11). Two-way ANOVA was performed to determine the interactions and main effects of training and ORX. **p < 0.01, ^(^*^)^*p* ≤ 0.10: main effect of training. ^(†)^*p* ≤ 0.10: main effect of ORX

**Fig. 11:**
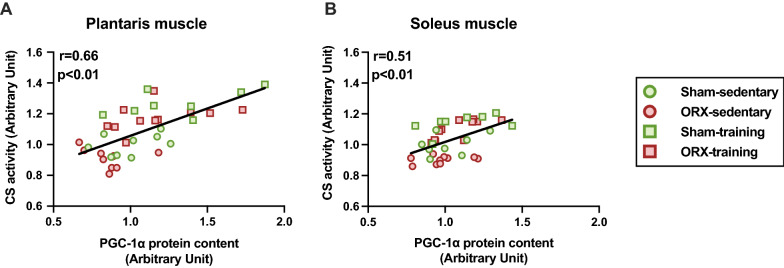
Correlations between CS activity and PGC-1α protein level. Correlation between CS activity and PGC-1α protein level in the plantaris (**A**) and soleus (**B**) muscles. Correlations between the two variables were studied using least-squares linear regression followed by Pearson’s correlation coefficient test

To better understand ORX-induced mitochondrial adaptation, we evaluated the protein levels of PGC-1α, a master regulator of mitochondrial biogenesis, and mitochondrial electron transport components. In the plantaris muscle, endurance training increased the protein levels of PGC-1α, NDUFB8, SDHB, UQCRC2, MTCO1, COXIV, and ATP5A (*p* < 0.01, Fig. 9A–G). ORX significantly decreased the protein levels of NDUFB8 (*p* < 0.01, Fig. 9B), UQCRC2 (*p* < 0.05, Fig. 9D), and COXIV (*p* < 0.01, Fig. 9G) in the plantaris muscle, providing additional support for the decrease in mitochondrial content. We also observed that ORX decreased PGC-1α protein levels in the plantaris muscle (*p* = 0.09, Fig. 9A). In the soleus muscle, endurance training significantly increased NDUFB8, MTCO1, and COXIV protein levels (*p* < 0.01, Fig. 10B, E and F) and tended to increase PGC-1α protein level (*p* = 0.06, Fig. 10A). ORX decreased the protein levels of NDUFB8 (*p* = 0.10, Fig. 10B) and COXIV (*p* = 0.10, Fig. 10F) in the soleus muscle. The protein levels of SDHB, UQCRC2, and ATP5A did not differ significantly (Fig. 10C, D and G). PGC-1α protein levels were positively correlated with CS activity in the plantaris (*p* < 0.01, *r* = 0.66; Fig. 11A) and soleus (*p* < 0.01, *r* = 0.51; Fig. 11B) muscles. Taken together, these results suggest that ORX reduces the mitochondrial content in skeletal muscle, and that changes in mitochondrial content can be attributed to changes in mitochondrial biogenesis.

### Metabolite transport proteins

**Fig. 12:**
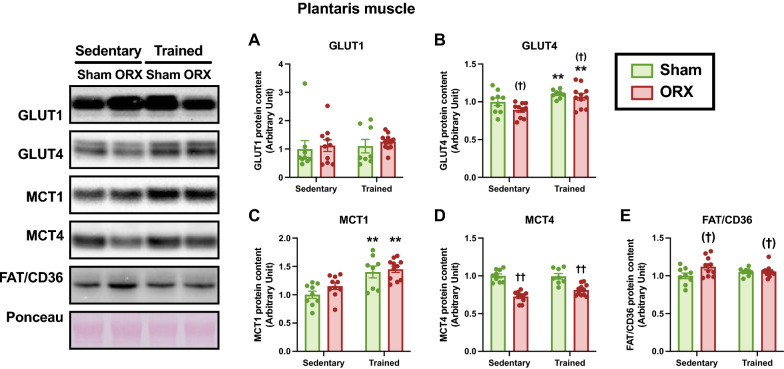
Metabolite transport protein levels in the plantaris muscle. Protein levels of glucose transporter (GLUT)-1 (**A**), GLUT4 (**B**), monocarboxylate transporter (MCT)-1 (**C**), MCT4 (**D**), and fatty acid translocase/cluster of differentiation 36 (FAT/CD36) (**E**) in the plantaris muscle. Data are expressed as the mean ± SEM (*n* = 8–11). Two-way ANOVA was performed to determine the interactions and main effects of training and ORX. **p < 0.01: main effect of training. ^††^*p* < 0.01, ^(†)^*p* ≤ 0.10: main effect of ORX

**Fig. 13:**
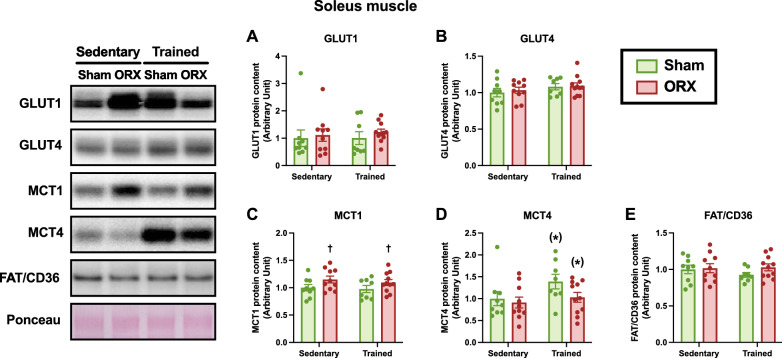
Metabolite transport protein levels in the soleus muscle. Protein levels of GLUT1 (**A**), GLUT4 (**B**), MCT1 (**C**), MCT4 (**D**), and FAT/CD36 (**E**) in the soleus muscle. Data are expressed as the mean ± SEM (*n* = 8–11). Two-way ANOVA was performed to determine the interactions and main effects of training and ORX. ^(^*^)^*p* ≤ 0.10: main effect of training. ^†^*p* < 0.05: main effect of ORX

Given that energy metabolism is facilitated by metabolite transport, we assessed the protein levels of key metabolite transporters. Endurance training significantly enhanced the protein levels of GLUT4 (*p* < 0.01, Fig. 12B) and MCT1 (*p* < 0.01, Fig. 12C) in the plantaris muscle. MCT4 protein level in the plantaris muscle was significantly decreased by ORX (*p* < 0.01, Fig. 12D). ORX tended to decrease GLUT4 protein level (*p* = 0.08, Fig. 12B) and increase FAT/CD36 protein level (*p* = 0.06, Fig. 12E) in the plantaris muscle. There was no significant difference in GLUT1 protein level in the plantaris muscle (Fig. 12A). In the soleus muscle, ORX significantly increased MCT1 protein level (*p* < 0.05, Fig. 13C). MCT4 protein level tended to increase after endurance training (*p* = 0.08, Fig. 13D). GLUT1, GLUT4, and FAT/CD36 protein levels did not differ in the soleus muscle (Fig. 13A, B and E). These results suggest that ORX induces muscle-specific changes in some metabolite transport proteins.

## Discussion

### Main findings and perspectives

The major finding of this study was that ORX-sedentary animals showed impaired exercise performance. We also observed that the blood lactate concentration after exercise was higher in the ORX-sedentary group than in the other three groups. Although blood lactate level can be determined by several factors (glycolysis, oxidation, circulation, etc.) [25], the present observation may suggest greater reliance on glycolytic metabolism. In addition, ORX decreased the glycogen content in the gastrocnemius muscle at rest. Enhanced glycolytic rate and small glycogen storage lead to rapid depletion of glycogen, which is a major factor responsible for fatigue [24, 26, 27]. Therefore, it is likely that the exercise intolerance observed in ORX-sedentary mice results from glycogen depletion due to greater reliance on glycogenolysis and low basal glycogen levels.

### Effects of ORX on glycolytic metabolism

The current observation of increased PFK activity in the plantaris muscle after ORX may underpin the notion that castrated animals rely heavily on glycogenolytic metabolism during exercise. In contrast to PFK activity, HK activity was decreased in the plantaris muscle of orchiectomized mice. PFK and HK are the rate-limiting enzymes in glycolysis [28]. PFK yields fructose-1,6-bisphosphate from fructose-6-phosphate derived from both glycogen and glucose, whereas HK catalyzes glucose phosphorylation. The maximal activity of PFK is reported to be much higher than that of HK [29]. These data indicate that PFK determines the overall glycolytic flux, especially glycogen breakdown rate, while HK is primarily involved in glucose metabolism. The partial knockout of HKII, a predominant isoform of HK, impairs exercise-stimulated muscle glucose uptake [30]. Exercise endurance capacity improves along with an increase in HKII protein content, which is related to muscle glucose uptake during exercise [31]. Furthermore, GLUT4 null mice exhibit exercise intolerance, reduced muscle glucose uptake, and increased muscle glycogen breakdown during exercise [32]. We observed that ORX significantly decreased HK activity and tended to decrease GLUT4 protein content in the plantaris muscle. Although measuring specific enzyme activity and protein levels is not always indicative of substrate flux in vivo, the present observations of the decrease in HK activity and GLUT4 protein content in the castrated animals may have resulted in diminished muscle glucose uptake, thereby reducing the glucose availability in the working muscle, leading to accelerated glycogen breakdown to maintain the overall glycolytic flux.

In the present study, the activity of LDH, which is involved in the conversion of pyruvate to lactate, was decreased by ORX in the plantaris muscle. This result seems to contradict the observation that blood lactate levels after exercise were elevated in the ORX sedentary group. LDH is highly abundant, and the catalysis of this enzyme is a near-equilibrium reaction [29, 33], suggesting that a decline in LDH activity does not affect the glycolytic capacity and lactate production. In addition to glycolytic enzyme activity, lactate transport activity across the plasmalemma via MCT1 and MCT4 influences the glycolytic capacity, as lactate efflux from the cells prevents end-product inhibition of glycolysis [34]. MCT1 is a high-affinity lactate transporter (Km = 3.5–8.3 mM) found greater quantity in oxidative fibers, whereas MCT4 is a low-affinity lactate transporter (Km = 25–32 mM) presented abundantly in glycolytic fibers [35]. On the basis of affinity for lactate and tissue-specific abundance, it is considered that MCT1 mainly facilitates lactate uptake, and that MCT4 primarily regulates lactate efflux [36]. In the current study, ORX decreased MCT4 protein level in the plantaris muscle. Since the post-exercise blood lactate level in the ORX-sedentary group (4.2 ± 1.1 mM) was much lower than the affinity of MCT4, we assumed that the muscle lactate level did not reach the value required for lactate release from skeletal muscle via MCT4. MCT1 was presumably sufficient to export lactate out of the skeletal muscle, as MCT can bidirectionally transport lactate across the sarcolemma depending on the concentration gradient [37].

HIF-1α is a transcription factor that regulates the expression levels of glycolytic enzymes and proteins [38], including HK, PFK, LDH, GLUT1, and MCT4. In the current study, the activities of HK and PFK, but not LDH, and protein levels of GLUT1 and MCT4 did not change concomitantly with HIF-1α protein levels. Besides HIF-1α, c-Myc also regulates glycolytic metabolism [39]. c-Myc mRNA expression is enhanced in castrated rat ventral prostate gland [40]. Moreover, nuclear receptor interacting protein 1 (NRIP1/RIP140) is another possible regulator of glycolysis [41]. In prostate cancer cells, RIP140 mRNA expression increases after androgen treatment [42]. Furthermore, a direct DNA binding site of the androgen receptor (AR) that promotes the transcription of glycolytic genes has been identified using chromatin immunoprecipitation with massively parallel DNA sequencing analyses [43]. Taken together, the coordinated transcriptional activity of several factors may explain the altered glycolytic enzyme activity and protein content observed in castrated animals.

### Effects of ORX on oxidative metabolism

Among the several parameters used as biomarkers of mitochondrial content, muscle CS activity is strongly correlated with mitochondrial content, as assessed by transmission electron microscopy [44]. In the present study, we found that ORX decreased CS activity in the plantaris and soleus muscles, suggesting that ORX decreases mitochondrial content in skeletal muscles. The current observations that ORX reduced the mitochondrial protein content in the plantaris muscle and β-HAD activity in the soleus muscle may provide additional support for this view. Under conditions of attenuated mitochondrial energy supply, glycolytic metabolism is activated to meet the energy requirement. It is, therefore, likely that the decline in mitochondrial content could be another factor contributing to the elevated blood lactate levels in ORX-sedentary animals.

The mitochondrial content depends on the balance between biogenesis and degradation (mitophagy) [45]. Androgen action via ARs regulates mitochondrial biogenesis. Activated AR complexes bind to the nuclear and mitochondrial gene response elements [46–48]. Another proposed pathway of androgen action is through the direct interaction of AR complexes with the androgen response elements of mitochondrial genes. Additional mechanisms may involve indirect interactions with the androgen response element in the nucleus to activate the transcription of genes encoding transcription factors, including PGC-1α, a master regulator of mitochondrial biogenesis [49]. According to a previous report, ORX-induced reduction in mitochondrial content is primarily attributed to altered biogenesis rather than mitophagy [50], as the authors observed concomitant changes in the mitochondrial protein content and mRNA and/or protein levels of PGC-1α. In support of this, we observed significant positive correlations between CS activity and PGC-1α protein levels in the plantaris and soleus muscles. Other researchers have reported that testosterone treatment increases PGC-1α protein levels and mitochondrial protein content in mouse skeletal muscles [51]. Collectively, it appears that androgens, particularly testosterone, alter the mitochondrial content in skeletal muscle by modulating mitochondrial biogenesis.

Ovariectomy-induced estrogen deficiency impairs the mitochondrial respiratory function in rodent skeletal muscle [52, 53], which implies that sex hormones affect mitochondrial respiratory functions. To the best of our knowledge, the role of androgens in mitochondrial respiratory function has yet to be clarified. Future studies are required to examine the ability of androgens to alter the mitochondrial respiratory function.

We and others have repeatedly shown that MCT1 protein level positively correlates with lactate uptake rate, proportion of slow-twitch oxidative fibers, and CS activity in skeletal muscle [54–57]. In addition, we previously reported concomitant changes in MCT1 protein level and CS activity in equine skeletal muscle during training and detraining [58]. Based on these observations, it is considered that MCT1 facilitates the transport of lactate into skeletal muscle for oxidation in mitochondria and that lactate uptake capacity changes together with muscle oxidative capacity. However, we observed here that ORX increased MCT1 protein level in the soleus muscle despite the decreased CS activity, suggesting a dissociation between lactate uptake and muscle oxidative capacity. We also observed elevated blood lactate concentrations in the ORX-sedentary group after exercise. Our data suggest the relative importance of muscle oxidative capacity compared with lactate uptake capacity in relation to blood lactate levels during endurance exercise. We previously demonstrated that PGC-1α overexpression enhances MCT1 protein level in rodent skeletal muscles [59, 60]. In the present study, PGC-1α protein level in the soleus muscle was unaltered by ORX, suggesting a PGC-1α independent increase in MCT1 protein levels. Further studies are required to clarify the factors and mechanisms that regulate MCT1 protein levels.

### ORX and hormonal regulation of substrate metabolism during exercise

Hormones, such as insulin, glucagon, and catecholamines (adrenaline and noradrenaline), exert strong influences on substrate metabolism [61]. During exercise, circulating insulin levels progressively decrease with time owing to adrenergic inhibition of pancreatic β-cells [62]. Previous studies showed a strong relationship between glucagon/insulin ratio and hepatic glucose output [63–65]. Although whether ORX changes glucagon/insulin ratio during exercise remains unclear, a previous study reported that ORX decreased basal glycogen concentration in the liver [66]. Hepatic glycogen was reported to be a key regulator of endurance capacity, because it is the primary storage for the maintenance of blood glucose level [67]. It is, therefore, possible that ORX diminishes glucose supply from the liver during prolonged exercise due to decreased basal levels of liver glycogen, which potentially impairs endurance performance. In addition to adrenergic stimulation, the decline in insulin level is also important for increasing adipose tissue lipolysis [68, 69], which leads to increased plasma fatty acid levels and skeletal muscle fat oxidation during exercise [70, 71]. Whether ORX changes hormonal secretions and responses, as well as substrate metabolism in other tissues during exercise requires further investigation.

### Muscle phenotype differences in adaptations to ORX

Given the current observation that ORX decreases mitochondrial enzyme activities and protein content in the plantaris and soleus muscles, it is likely that mitochondrial adaptation to ORX is not muscle phenotype-specific. In the present study, changes in glycolytic enzyme activities in the plantaris muscle appeared to be more sensitive to ORX than those in the soleus muscle. This observation may be explained by the greater affinity [8] and/or number [9] of ARs in fast-twitch muscle fibers, because the plantaris muscle comprises more than 90% of fast-twitch fibers, whereas the soleus muscle comprises around 50% of slow-twitch fibers [72]. It should be noted that some human skeletal muscles are composed of more than 50% of slow-twitch fibers [73, 74]. Whether the present findings are also the case for human muscle should be clarified in the future study.

### Effects of endurance training

High-intensity interval training (HIIT) is known to enhance the glycolytic capacity owing to increased levels of HK, PFK, LDH, and MCT4 [75–79]. We previously reported that HIIT increased HIF-1α protein level in mouse skeletal muscle [80], suggesting that HIF-1α may be accountable, in part, for HIIT-induced alterations in glycolytic capacity. In the present study, we found that endurance training enhanced HK activity in the plantaris and soleus muscles and PFK activity in the soleus muscle, even though HIF-1α protein levels were decreased in the plantaris muscle and did not change in the soleus muscle. These observations suggest that HIF-1α is not solely responsible for glycolytic adaptation to HIIT and that other transcriptional factors may be involved in endurance training-induced changes in glycolytic enzyme activity and protein levels.

Mitochondria are fundamental cellular components that are related to health and disease [81]. In the current study, although ORX decreased the mitochondrial enzyme activity and protein levels in skeletal muscles, which were enhanced by endurance training. In addition, the PGC-1α protein level after endurance training was increased in the plantaris muscle and tended to increase in the soleus muscle. These observations suggest that the increase in mitochondrial content after endurance training may stem, in part, from PGC-1α protein abundance, and that endurance training would be a viable approach to counteract hypogonadism-induced decline in mitochondrial content and the development of adverse health outcomes. Moreover, the increase in mitochondrial content contributes to endurance performance [82], as it enables the skeletal muscle to spare glycogen during exercise [27]. We found that endurance training restored the exercise performance and normalized post-exercise blood lactate levels in castrated animals. Since endurance training did not decrease HK and PFK activities in the skeletal muscles, the effects of endurance training may be ascribed primarily to the increased mitochondrial content.

### Vascular adaptations to ORX and endurance training

Skeletal muscle capillarity is also an important factor contributing to endurance performance [83]. In the present study, although we were unable to assess capillarity, the protein content of HIF-1α, which is a transcription factor inducing angiogenesis [84], was declined by ORX. A previous report demonstrated that ORX decreased in vivo angiogenesis, resulting in the reduced capillary density and blood vessel diameter in mouse hindlimbs [85]. Another study reported that the critical power, measured in units of power rather than speed, was positively related to skeletal muscle capillarity in endurance-trained humans [86]. We, therefore, assume that ORX-induced decline in vascularity decreased the critical speed of castrated animals, resulting in impaired performance.

Together with mitochondrial biogenesis, endurance training induces angiogenesis in skeletal muscle [87, 88]. In the present report, however, HIF-1α protein content after endurance training was not restored in the soleus muscle and decreased in the plantaris muscle. Other investigators reported that accumulation of HIF-1α by knocking down prolyl hydroxylase domain protein (PHD) 2, which hydroxylates HIF-1α for ubiquitination and degradation, increased capillaries in mouse skeletal muscle without exercise training [89]. They also showed that endurance training increased capillary density to the same extent in the PHD2 knockdown and wild-type animals [89]. These data may suggest that HIF-1α can increase capillaries in the skeletal muscle, but exercise training-induced angiogenesis is not predominantly mediated through HIF-1α. Another pathway involved in angiogenesis is PGC-1α [90, 91]. It has previously been demonstrated that PGC-1α transgenic mice display the increased capillary density and capillary-to-fiber ratio in skeletal muscle together with fatigue resistance to tetanic muscle contraction [92], although PGC-1α overexpression also increases mitochondrial content in the skeletal muscle [93]. In the current investigation, PGC-1α protein content after endurance training significantly increased in the plantaris muscle, and tended to increase in the soleus muscle. These observations may suggest that endurance training enhances skeletal muscle vascularity through the PGC-1α-dependent pathway, leading to less fatiguability. Finally, cross-sectional data showed that high-intensity training was more effective for increasing the capillary-to-fiber ratio, compared to lower intensities of exercise training [94]. It is, therefore, worth comparing endurance training with high-intensity training to develop effective training strategies for patients with androgen insufficiency or deficiency.

## Conclusions

This study demonstrated that endurance training restores ORX-induced impaired physical performance. This effect seems to be attributable to enhanced mitochondrial content, as endurance training did not normalize the ORX-induced changes in glycolytic enzyme activity. We conclude that endurance training may be a potential alternative to androgen replacement therapy for alleviating the negative metabolic consequences of hypoandrogenism.

## Data Availability

The data supporting the findings of this study are available from the corresponding author upon reasonable request.

## Abbreviations

β-HAD: β-Hydroxyacyl-CoA dehydrogenase
ANOVA: Analysis of variance
AR: Androgen receptor
ATP5F1A/ATP5A: ATP synthase F1 subunit alpha
COX: Cytochrome *c* oxidase
CPT: Carnitine palmitoyltransferase
CS: Citrate synthase
GLUT1: Glucose transporter 1
HIF-1α: Hypoxia-inducible factor 1-α
HIIT: High-intensity interval training
HK: Hexokinase
LDH: Lactate dehydrogenase
MCT1: Monocarboxylate transporter 1
MTCO1: Mitochondrially encoded cytochrome c oxidase 1
NDUFB8: NADH: ubiquinone oxidoreductase subunit B8
NRIP1/RIP140: Nuclear receptor interacting protein 1
ORX: Orchiectomy
PFK: Phosphofructokinase
PGC-1α: Peroxisome proliferator-activated receptor γ coactivator 1-α
PHD: Prolyl hydroxylase domain protein
RER: Respiratory exchange ratio
SDHB: Succinate dehydrogenase complex iron sulfur subunit B
TG: Triglyceride
UQCRC2: Ubiquinol-cytochrome *c* reductase core protein 2
VCO_2_: Carbon dioxide production
VO_2_: Oxygen consumption
